# Factors relating to failure to quit smoking: a prospective cohort study

**DOI:** 10.1590/S1516-31802011000600003

**Published:** 2011-12-01

**Authors:** Renata Cruz Soares de Azevedo, Rejane Firmino Fernandes

**Affiliations:** I MD, PhD. Psychiatrist and Professor of the Department of Medical Psychology and Psychiatry, Faculdade de Ciências Médicas, Universidade Estadual de Campinas (FCM-Unicamp), Campinas, São Paulo, Brazil.; II MSc. Clinical Psychologist. Graduate Program in Medical Sciences, Faculdade de Ciências Médicas, Universidade Estadual de Campinas (FCM-Unicamp), Campinas, São Paulo, Brazil.

**Keywords:** Smoking, Therapeutics, Treatment outcome, Tobacco use cessation, Ambulatory care, Tabagismo, Terapêutica, Resultado de tratamento, Abandono do uso de tabaco, Assistência ambulatorial

## Abstract

**CONTEXT AND OBJECTIVE:**

Considering the difficulties in stopping smoking, this article aimed to identify factors relating to failure of attempts to quit smoking among smokers who sought care at an outpatient clinic in a general university hospital.

**DESIGN AND SETTING:**

Prospective cohort study evaluating 100 smokers who sought treatment at the Psychoactive Substances Outpatient Clinic.

**METHODS:**

The variables gathered were sociodemographic factors; degree of dependence (Fagerström questionnaire); stage of motivation for change (University of Rhode Island Change Assessment Scale); and presence of depression and anxiety (Hospital Anxiety and Depression Scale). The patients were followed up after 4, 8, 12 and 24 weeks to identify factors relating to failure to quit smoking.

**RESULTS:**

The patients were mostly women (75%), between 40 and 59 years of age (67%); with incomplete elementary education (60%); with leisure activities (57%); suffering from tobacco-related disease (53%); with previous attempts to quit smoking (70%); with a medical recommendation to stop (51%); with encouragement to stop (66%); and with a high degree of dependence (78%). The main motivational stage was contemplation/action (43%); the anxiety rate was 64% and the depression rate was 39%. The quitting rate was 66% among adherents and 17% among non-adherents (P < 0.001). Lack of success was correlated with absence of leisure, higher education and absence of tobacco-related disease.

**CONCLUSION:**

The variables of lack of leisure activities, higher education and/or lack of tobacco-related disease correlated with failure to quit smoking among smokers who sought treatment at an outpatient clinic in a tertiary general hospital.

## RESUMO

**CONTEXTO E OBJETIVO:**

Considerando as dificuldades na cessação do tabagismo, este artigo objetiva identificar fatores relacionados ao insucesso na tentativa de cessação tabágica de fumantes que procuraram atendimento em ambulatório de um hospital geral universitário.

**TIPO DE ESTUDO E LOCAL:**

Estudo de coorte prospectivo, que avaliou 100 fumantes que procuraram tratamento no Ambulatório de Substâncias Psicoativas.

**MÉTODOS:**

Avaliaram-se variáveis sociodemográficas, grau de dependência (Questionário de Fagerström), estágio de motivação para a mudança (University of Rhode Island Change Assessment), presença de depressão e ansiedade (Hospital Anxiety and Depression Scale). Os pacientes foram acompanhados em 4, 8, 12 e 24 semanas para identificar fatores relacionados ao insucesso na cessação do tabagismo.

**RESULTADOS:**

Os pacientes eram, na maioria, mulheres (75%), entre 40 e 59 anos de idade (67%), com ensino fundamental incompleto (60%), com atividades de lazer (57%), portadores de doença tabaco-relacionada (DTR) (53%), com tentativas anteriores de parar de fumar (70%), que receberam recomendação médica (51%) e incentivo para a cessação (66%) e com elevado grau de dependência (78%). O estágio de motivação principal foi contemplação/ação (43%), a taxa de ansiedade foi de 64% e de depressão, 39%. A taxa de cessação foi de 66% entre os que aderiram e de 17% entre aqueles que não aderiram (P < 0,001). O insucesso foi relacionado à ausência de lazer, maior escolaridade e ausência de DTR.

**CONCLUSÃO:**

Falta de atividades de lazer, maior nível educacional e/ou não ter DTR foram variáveis relacionadas ao insucesso na cessação tabágica para tabagistas que procuram tratamento em ambulatório inserido em hospital geral terciário.

## INTRODUCTION

Worldwide, it has been estimated that there are 1.3 billion smokers and that 80% of them live in developing countries.^1^ Consequently, more than half of the world's population is exposed, directly or indirectly, to the harmful effects of nicotine and other toxic substances in tobacco. According to data from the World Health Organization (WHO), approximately 700 million children are involuntarily exposed to tobacco smoke, especially within the home environment, thus increasing the pneumonia and bronchitis rates and the risk of sudden death, among other diseases.^2^ Smoking is the leading cause of preventable death worldwide and is considered to be a serious public health problem. In 2009, the WHO estimated that five million deaths were attributable to smoking. If current patterns of tobacco use are not reversed or the measures provided for global tobacco control are not adopted, the projection for 2025 is 10 million deaths.^1^

In Brazil, smoking affects 17.2% of the population over 15 years of age.^3^ Data from the National Cancer Institute indicate rates of 200,000 deaths per year, i.e. 23 people die every hour because of smoking-related conditions.^4^

Despite the dissemination of information regarding the dangers of smoking, there are several difficulties in the process of breaking an addiction, going from the decision to quit to success and persistence in stopping. This difficulty is due to several mechanisms, including positive reinforcement (because the action of nicotine on the central nervous system results in feelings of pleasure, increased disposition, attention and reduced appetite), conditioning (triggered by environmental stimuli and positive and negative emotions associated with smoking) and negative reinforcement (sustained use to avoid the discomfort of withdrawal syndrome symptoms, especially anxiety, dysphoria, increased appetite, irritability and difficulty in concentrating).^5^

The effect of nicotine on the brain and the pharmacological and behavioral processes that determine nicotine addiction contribute towards the difficulty in maintaining abstinence. The symptoms of nicotine withdrawal are the primary reason why only 5% to 10% of smokers manage to quit without help.^6^

The variables associated with success in stopping smoking should be considered in strategies for approaching smokers. Several studies^7^ have shown that older men and women, those who are married and those with high socioeconomic status all display better results relating to stopping smoking. Moreover, the number of cigarettes smoked per day is also directly related to better responses towards attempts to quit smoking.

To optimize the strategies for treating smoking, it is also relevant to discuss the factors related to failure in treating smokers. Studies^7,8^ have shown that high levels of anxiety and depression in patients make adherence to and success in treating smoking addiction quite difficult, and that starting smoking at an early age decreases the likelihood of adherence to treatment.

## OBJECTIVE

To identify factors relating to failure in attempts to quit smoking among smokers who sought medical care at an outpatient clinic in a general university hospital.

## METHODS

This was a quantitative, descriptive and prospective cohort study that assessed smokers who sought treatment for the first time at a specialized clinic in a public university. The subjects were evaluated at the time of their first attendance at the clinic and were reassessed after 4, 8, 12 and 24 weeks.

The study used convenience sampling, with inclusion of 100 individuals who visited the Psychoactive Substances Outpatient Clinic (Ambulatório de Substâncias Psicoativas, ASPA) of the Clinical Hospital, Universidade Estadual de Campinas (HC/Unicamp) for the first time between March 2008 and March 2009, in order to receive treatment for tobacco addiction.

The inclusion criteria were that the patients could be either male or female and that they needed to be over 18 years of age, to have been daily smokers for at least six months, to be seeking treatment for stopping smoking at ASPA/Unicamp for the first time and to have sufficient time to be interviewed before participating in the initial care at the clinic.

The exclusion criteria were the presence of any cognitive or psychological impairment that prevented understanding of the research and/or non-acceptance of the informed consent statement.

Every patient who comes to ASPA because of smoking problems is routed to a motivation group, which functions as an open group, without the need for a formal referral. The group meetings for outpatients are held weekly, conducted by the outpatient professional team (psychiatrist, pulmonologist, dentist, nurse, social worker, psychologist or occupational therapist) and observed by postgraduate students, residents and public health providers under training. The group meetings are one hour in length and the topics of discussion include the difficulties in quitting, mechanisms relating to nicotine addiction, barriers against stopping, treatment alternatives and strategies for recognizing personal motivators for stopping smoking. After a patient has participated in at least four group sessions and is motivated to move forward in the process, he/she is instructed to complete an individual assessment prior to inclusion in the therapeutic group.

We used two semi-structured questionnaires (one for the initial assessment and one for the follow-up at weeks 4, 8, 12 and 24) and five psychometric instruments, all applied individually by the first author of the study, as described below.

- Initial assessment file: this included a survey of sociodemographic data (gender, age, employment status, education level, origin, marital status, children, age of the children, people who the patient was living with, leisure activities, religion and religious practice), clinical data (whether the patient had any physical illnesses and what type; mental illnesses and what type; whether the patient was consuming alcohol and how often; and what use was made of other psychoactive substances) and data relating to the recommendation to come to the clinic.
- Fagerström addiction questionnaire:^9,10^ this consisted of six questions that evaluated tobacco addiction, with scores ranging from 0 to 10. These scores were used to classify dependence as low, medium or high.
- University of Rhode Island Change Assessment Scale (URICA):^11,12^ this scale evaluated the stage of action for change. It consists of 32 items divided into four subscales: precontemplation, contemplation, action and maintenance. This scale can be used to evaluate any addictive behavior, and has previously been validated and adapted for the Brazilian population.
- Hospital Anxiety and Depression Scale (HAD):^13,14^ this scale was primarily designed to detect mild degrees of depression and anxiety among non-psychiatric patients in a general hospital. Patients are asked to answer based on how they felt during the preceding week. In the current study, a cutoff value of 8/9 was used.
- Semi-structured questionnaire for data on smoking: this investigated the history of tobacco use; whether the patient was living with smokers; whether the patient had had any tobacco-related health problems; data on previous attempts to quit, i.e. whether it had previously been attempted and how often it had been attempted; and data on motivation to stop smoking, i.e. whether there had been any encouragement, who had provided the main encouragement, the family's reaction to the decision to stop and what motivated the decision to stop smoking.
- Follow-up questionnaire: a semi-structured questionnaire was applied in the 4^th^, 8^th^, 12^th^ and 24^th^ weeks after the first interview. The questionnaire investigated patient attendance at care services, adherence or non-adherence to treatment and outcome (i.e. whether the patient stopped, reduced, increased or maintained smoking, or relapsed).

We also identified tobacco-related comorbidities that were mentioned by patients: cardiovascular diseases (hypertension, atherosclerosis, angina, myocardial infarction, stroke and thromboangiitis obliterans), respiratory conditions (chronic bronchitis, emphysema, pneumothorax, congestive cardiorespiratory, asthma and respiratory infections) and cancers (in the lungs, mouth, tongue, pharynx, larynx, esophagus, stomach, pancreas, liver, bladder, colorectum, prostate, skin and lymphoid cells).^15^

Patients who attended the motivational group meetings but did not begin attending the therapeutic group meetings were deemed to present non-adherence to treatment. Patients who had not stopped smoking at follow-up assessments (weeks 4, 8, 12 and 24) were deemed to present therapeutic failure. The results presented in this article refer to the outcomes evaluated at the end of the 24^th^ week of the follow-up. The criterion for adherence to treatment was preestablished: patients were considered to be adherent to treatment when they attended the motivational group sessions at least four times.

Patients who sought ASPA to obtain treatment for tobacco addiction during the study period and who arrived at the clinic at least 20 minutes prior to the group motivational session were asked whether this was their first time seeking this service. When the answer was affirmative, they were invited to participate in the study through providing them with an explicit description of its goals and allowing them to read the informed consent statement. Those who did not arrive for the first day of treatment early enough to be interviewed prior to the group meeting were excluded. The aim in choosing not to include in the study patients who were not seeking ASPA for the first time and not to conduct interviews after the group motivational session was to avoid using terms that patients in this group had learned and to avoid situations in which the patients had learned not to express their spontaneous motives. For patients who were included in the survey, after they had agreed to participate, the aforementioned tools were administered.

To perform the follow-up, the researcher contacted the patients in an outpatient setting at the end of the visits. When the patient did not attend the clinic on the scheduled day of their follow-up interview (weeks 4, 8, 12 or 24), telephone calls were made to ask them to come to the clinic during the following week. If telephone contact was not possible, the following measures were used: telephone directory aided by Internet search; and assistance from the Social Services of the Clinical Hospital of Unicamp. After three requests for attendance, if the patient did not visit the clinic, a follow-up interview was conducted by telephone. If both measures were unsuccessful, the patient was considered to have been lost from the follow-up.

This study was approved by the Research Ethics Committee of Unicamp under registration number 0690.0.146.000-07.

The data gathered were entered into the SAS software database (SAS – Statistical Analysis Systems, version 9.1.3., 2002-2003). To analyze the relationship between categorical variables, the chi-square test or the Fisher exact test (for expected values lower than 5) was used. To analyze non-adherence and therapeutic failure outcomes, the Cochran and Friedman tests were used. To study the factors associated with treatment failure at week 24, logistic regression analyses, univariate models and multivariate models with stepwise criteria of variable selection were used. The significance level for statistical tests was 5% (P < 0.05).

## RESULTS

**Figure 1.**
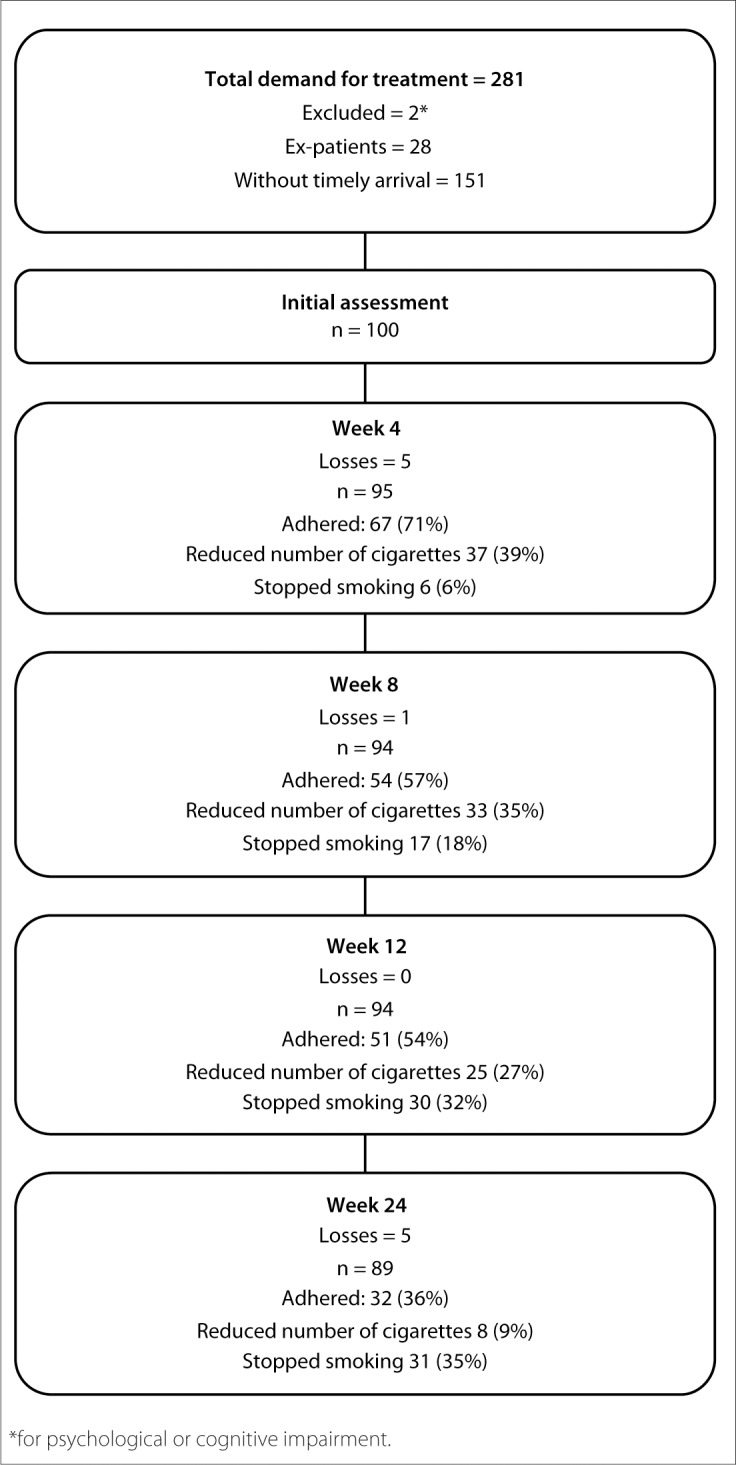
Monitoring of patients during the study.

During the study period, 281 people sought treatment for tobacco addiction at the Psychoactive Substances Outpatient Clinic of the Clinical Hospital, Unicamp. Of these, 151 people did not arrive for the first day of treatment sufficiently early to be interviewed prior to the group meeting and were not included in the study. Furthermore, 28 subjects had previously been patients at the clinic and were making a new attempt because of a relapse, and were therefore excluded. Two subjects were excluded due to psychological or cognitive impairment: one showing significant cognitive impairment and the other showing symptoms of intoxication during the interview. Thus, we evaluated 100 smokers who sought treatment to stop smoking for the first time at ASPA (Figure 1).

There was a loss of 11% of the patients over the subsequent six months. The highest rates of loss were concentrated within the first and fourth weeks (5%) and from the 12^th^ to the 24^th^ week (5%).

Of the smokers evaluated in this study, the majority (64%) had begun smoking before reaching 15 years of age and 72% had smoked for 30 years or more.

Other data relating to the patients’ profile are shown in Table 1.

**Table 1.** Profile of the study population (n = 100)

Variable	n	%
Sex		
Female	75	75%
Male	25	25%
Age group		
≤ 39 years	21	21%
40-59 years	67	67%
≥ 60 years	12	12%
Educational level		
≤ Elementary uncompleted	60	60%
≥ Elementary complete	27	27%
Professional status		
Working	53	53%
Off work because of illness	20	20%
Retired due to illness	11	11%
Unemployed	9	9%
Marital status		
With partner	54	54%
Without partner	46	46%
Living with smoker	43	43%
With leisure activities	57	57%
With tobacco-related disease (n = 53)		
Respiratory	30	55%
Cardiovascular	13	24%
Cancer	10	18%
Attempted to quit previously	70	70%
Recommendation		
Doctor	51	51%
Others	44	44%
Fagerström		
High	78	78%
Medium or low	22	22%
Received encouragement to quit	66	66%
URICA		
Contemplation	36	36%
Contemplation/action	43	43%
Action	21	21%
HAD anxiety-positive	64	64%
HAD depression-positive	39	39%
Main reasons for non-adherence (n = 57)		
Schedule difficulty	27	47%
Personal problems	6	10%
Transport	5	9%
Lack of interest	5	9%

URICA = University of Rhode Island Change Assessment; HAD = Hospital Anxiety and Depression Scale

Table 2 presents the profiles of the patients who succeeded in stopping and failed to stop smoking after six months.

**Table 2.** Profile of failure and success regarding quitting smoking

Variable	n	Failure (%)	Success (%)	P
Total	89*	58 (65)	31 (35)	
Sex				
Female	67	43 (74)	24 (77)	0.7
Male	22	15 (26)	7 (23)
Age group				
< 50 years	45	30 (52)	15 (48)	0.7
≥ 50 years	44	28 (48)	16 (52)
Schooling				
≤ Elementary uncompleted	67	40 (69)	27 (87)	0.05
≥ Elementary complete	22	18 (31)	4 (13)
Professional status				
Working	48	33 (57)	15 (48)	0.4
Unemployed	41	25 (43)	16 (52)
Marital status				
With partner	48	31 (53)	17 (55)	0.9
Without partner	41	27 (47)	14 (45)
Living with smoker				
Yes	39	28 (48)	11 (35)	0.2
No	50	30 (52)	20 (65)
Leisure activities				
With leisure activities	50	28 (48)	22 (71)	0.04
Without leisure activities	39	30 (52)	9 (29)	
Tobacco-related disease (n = 53)				
With tobacco-related disease	48	26 (45)	22 (71)	0.01
Without tobacco-related disease	41	32 (55)	9 (29)
Attempted to quit previously				
Yes	61	38 (66)	23 (74)	0.4
No	28	20 (34)	8 (26)
Recommendation				
Doctor	49	31 (53)	18 (58)	0.1
Others	40	27 (47)	13 (42)
Fagerström				
High	68	44 (76)	24 (77)	0.8
Medium or low	21	14 (24)	7 (23)
Received encouragement to quit	60	41(71)	19 (61)	0.3
URICA				
Contemplation	32	23 (40)	9 (29)	0.3
Contemplation/action	38	25 (43)	13 (42)
Action	19	10 (17)	9 (29)
HAD anxiety-positive	58	38 (66)	20 (64)	0.9
HAD depression-positive	35	24 (59)	11 (35)	0.5

* Losses over the course of the monitoring (n = 11).

The quitting rate was 66% among those who adhered to the treatment and 17% among those who did not adhere (P < 0.001). The criterion for compliance was at least four weeks in group motivational sessions. The reasons given for noncompliance were often difficulty in finding time (27% of the patients), difficulty in obtaining transportation (5%) or personal health problems (8%).

The variables included in logistic regression analysis on the failure to quit smoking were gender, age, education level, professional status, marital status, living with smoker, leisure activities, religion, religious practice, tobacco-related disease, Fagerström, URICA, HAD for depression and anxiety and main encouragement.

The results from multivariate logistic regression analysis indicated that the variables of tobacco-related disease, leisure and education level were significantly associated with treatment failure at 24 weeks. Moreover, the subjects with a high risk of failure were those without tobacco-related disease (2.7 times), without leisure (3.9 times) and with higher education (4.5 times) (Table 3).

**Table 3.** Multiple regression analysis on factors for failure to quit smoking at 24 weeks

Variable	P	OR	95% CI
Leisure			
Yes	0.010	3.91	1.39-11.01
No			
Education			
≤ Elementary	0.024	4.51	1.22-16.58
> Elementary			
Tobacco-related disease			
Yes	0.049	2.70	1.01-7.25
No			

OR = odds ratio; CI = confidence interval.

## DISCUSSION

The main objective of this study was to investigate factors associated with failure to quit smoking, among a group of smokers who sought treatment at a specialized clinic. Our intention was to contribute towards increasing the quitting rate based on identifying factors that influence the failure to quit smoking.

The sociodemographic profile of the smokers who sought treatment was that they were mostly women, within the age range of 40 to 59 years; they lacked complete elementary education, were employed, were Catholic and had a partner. This is compatible with data from the Brazilian literature.^16^ These data suggest that studies addressing two aspects of treating tobacco addiction are important: the reasons why lower rates of men seek treatment (even though the prevalence of smoking is higher among males); and the need to develop strategies to raise smokers’ awareness that they should seek, and health professionals’ awareness that they should recommend, treatment at an earlier age. Through this, the harmful consequences relating to long-term exposure to cigarette smoke may be minimized.

In terms of smoking history, most of the patients in this study started the habit at 15 years of age or younger, had smoked for 30 years or more and had a high degree of addiction according to the Fagerström test. These data are consistent with other work in this field.^17^ Moreover, most of the patients had previously attempted to quit smoking and had been encouraged to do so and seek treatment. This may indicate that this was a subgroup of patients with greater chances of success.^7^

The data from this study emphasize the harmful consequences of smoking,^15^ such as the high rates of tobacco-related disease, with prevalence of respiratory diseases. Furthermore, approximately one-third of the patients were off work or had retired due to illness. Moreover, it is possible that because of their diseases, such individuals may have received more encouragement from physicians and other health professionals to stop smoking because they used greater quantities of health services in addressing their health problems.^18^

The comorbidity rates relating to depression and anxiety, evaluated using the HAD Scale (39% and 64%, respectively), were higher than the rates for the population in general.^19^ These variables were not associated with the outcome studied, probably because of the limitations imposed by the number of subjects. However, it is important for these comorbidities to be assessed by healthcare professionals, with the aim of achieving a broader approach to tobacco addiction.

One study^20^ has suggested that lower rates of compliance with treatment are directly associated with lower rates of abstinence from smoking. In the current study, success in stopping smoking was significantly higher among those who adhered to the treatment (66% versus 17% among non-adherents). The relationship between adherence and success indicates the importance of studies that contribute towards optimizing the adherence strategies. Moreover, this finding suggests that there is a need for studies to deepen the understanding of success rates higher than the spontaneous quitting rate (around 5% to 10%),^6^ even among patients who do not adhere to treatment.

The quitting rate of 35% in this study is similar to the findings in other Brazilian and international studies.^17,21^ This result should encourage expansion of services for smokers, so as to contribute towards reducing the morbidity-mortality associated with tobacco.

One factor that was related to treatment failure in the multivariate analysis was the lack of leisure activities. It was shown in one study^22^ that participation in meetings outside of the workplace, at church or with family members was predictive of success among daily smokers, thus suggesting that social participation promotes successful treatment for smokers. Another study^18^ reported that smokers who engaged in other activities in their routine and did not need to smoke to pass the time had greater success in the treatment. This indicates that there is a need to integrate strategies considering this variable, in order to facilitate stopping smoking.

The second noteworthy factor associated with failure was a higher education level. This finding was not corroborated by other studies,^23^ which showed an inverse relationship. It is likely that this finding relates to the fact that the present study evaluated individuals who sought treatment at a public university hospital with a care profile characterized by high demand, limited space and participation of students and other professionals in training as observers. In addition, the language adopted in the groups indicated that most participants in this study had a low educational profile. However, other studies, particularly among Brazilian populations, should be undertaken in order to examine this hypothesis.

One factor cited in the multivariate analysis as associated with failure to quit smoking was the absence of tobacco-related disease. A recent study^24^ noted that the presence of tobacco-related disease was correlated with seeking treatment and presenting greater motivation to quit. From these data, it can be suggested that smokers could be targeted with greater emphasis on the benefits associated with prevention of tobacco-related disease, as well as the quality-of-life benefits from stopping smoking, even before the emergence of any disease related to smoking.

Some limitations of this study should be highlighted. The first relates to the small number of subjects evaluated, which limited the power of statistical analysis. Another limitation was the non-inclusion of individuals who did not arrive on the first day with sufficient time to undergo the interview before their participation in the host group. Although this method prevented us from including a significant number of smokers in the study, it was used because we considered it to be more important for the patients not to be compromised in their search for treatment (i.e. by not starting their care on the desired day) as a result of our research project. A further limitation was that stopping smoking was measured through self-reporting, without biological validation such as serum cotinine measurement. Finally, it is important to mention that the study was conducted using a convenience sample (a population that sought treatment with voluntary compliance), which imposes limitations on extrapolating the data to different populations.

## CONCLUSION

The variables of lack of leisure activities, higher education and/or absence of tobacco-related disease were correlated with failure to quit smoking, among smokers who sought medical care at an outpatient clinic in a tertiary general hospital.
